# Insulin degludec/liraglutide versus its monotherapy on T2D patients: A lifetime cost-utility analysis in China

**DOI:** 10.3389/fphar.2022.1011624

**Published:** 2022-11-18

**Authors:** Guangxin Han, Shanshan Hu, Xiaoning Zhang, Zhikun Qiu, Zhe Huang

**Affiliations:** ^1^ School of Business Administration, Shenyang Pharmaceutical University, Shenyang, China; ^2^ Department of Clinical Pharmacy, The First Affiliated Hospital of Guangdong Pharmaceutical University, Guangzhou, Guangdong, China; ^3^ Department of Clinical Pharmacy, Shanghai General Hospital, Shanghai Jiao Tong University School of Medicine, Shanghai, China; ^4^ Department of Thoracic Surgery, Nanfang Hospital, Southern Medical University, Guangzhou, Guangdong, China

**Keywords:** cost-utility analysis, type 2 diabetes, IDegLira, model simulation, UKPDS OM2

## Abstract

**Introduction:** IDegLira (brand name Xultophy) is a novel fixed ratio combination of insulin degludec and liraglutide for type 2 diabetes (T2D) patients. This study aimed to investigate the lifetime cost-effective value of IDegLira compared with its single component (Degludec or Liraglutide) and to explore the suitable annual cost of IDegLira if necessary.

**Methods:** UKPDS OM2 was applied to determine the long-term quality-adjusted life years (QALYs) and total costs. The efficacy data that were inputted into the model were synthesized from 6 randomized clinical trials (RCTs) that directly assessed the clinical benefit of IDegLira and its components in the treatment of uncontrolled T2D patients. The economic results were examined by one-way sensitivity analysis (OSA) and probabilistic sensitivity analysis (PSA). Further price reduction of IDegLira was investigated by binary search.

**Results:** The IDegLira, IDeg, and Lira yielded 11.79 QALYs, 11.62 QALYs, and 11.73 QALYs and total cost of $20281.61, $3726.76, and $11941.26, respectively. The incremental cost-utility ratio (ICUR) of IDegLira versus IDeg was $99464.12/QALYs, and the ICUR of IDegLira versus Lira was $143348.26/QALYs, which indicated that IDegLira was not a cost-effective therapy for T2D patients compared with its components at the current price from a Chinese national healthcare system perspective. Base case results were robust to OSA and PSA. A further binary search showed that IDegLira appears to only be cost-effective if the annual cost of IDegLira is decreased by 58% when IDeg is considered as a reference, or by 30.57% when Lira is considered as a reference.

**Conclusion:** In conclusion, IDegLira appears to not be cost-effective when compared with the current prices of IDeg or Lira for T2D patients in China. However, after the binary search, IDegLira appears to only be cost-effective if the annual cost of IDegLira is decreased 58% when IDeg is considered as a reference, or by 30.57% when Lira is considered as a reference.

## Introduction

Diabetes is becoming an increasingly urgent global public health problem. In 2021, it was reported that approximately 537 million adults worldwide have diabetes, and this number will increase to 643 million in 2030 and 783 million in 2045. The increasing global prevalence of diabetes appears to be related to the growing and aging population, which is an ongoing issue today (Shi et al., 2021). The International Diabetes Federation (IDF) Diabetes Atlas reported that the burden of diabetes is increasing (Sun et al., 2022) and that the condition caused 6.7 million deaths in 2021. Moreover, global health expenditure related to diabetes account for at least 966 billion US dollars, which is an increase of more than 316% in the last 15 years (Sun et al., 2022). China has the largest number of diabetes cases, and the prevalence of diabetes increased from 10.9% in 2013 to 12.4% in 2018 (Wang et al., 2021). In the last decade, diabetes become an epidemic in China (Xu et al., 2013), with 63.3% of cases being undiagnosed. However, the situation is still not optimistic among people who realize they have diabetes. Approximately 32.9% of patients with diabetes are treated with medicine, and adequate control is achieved in 50.1% of diabetes patients who receive treatment (Wang et al., 2021). The costs of diabetes management and diabetes-related complications is significations for patients with diabetes. In 2014, the largest diabetes burden of $170 billion was recorded for China, followed with $105 billion for United States, $73 billion for India, and $37 billion for Japan (2016). About one-third (32.2%) of people with diabetes will eventually develop diabetic cardiovascular disease (CVD), including stroke, angina, heart failure, and myocardial infarction (MI). CVD accounted for 20.1% of all death in people without diabetes, for 47.2% of all death in people with diabetes (Einarson et al., 2018). Hence, diabetes is a heavy socioeconomic burden on people with diabetes, their families, and the country. Therefore, the availability of cost-effective therapies is becoming increasingly important in China.

More than 90 of diabetes patients are diagnosed with type 2 diabetes (T2D). T2D is characterized by chronic progression and progressive beta cell damage, which requires treatment across the life span. Diabetes is an expensive and harmful disease, and personalized self-management of the condition depends on multidimensional treatments and has become essential for patients to achieve management goals. Diabetes managements initially starts with a healthy diet, regular exercise, and body weight management, followed by the addition of oral hypoglycemic drugs (OADs) and finally injectable hypoglycemic drugs. Although most diabetes cases eventually need insulin therapy, the initiation of insulin is often delayed (Inzucchi et al., 2015; Khunti and Millar-Jones, 2017). The reasons for delay of insulin initiation include concerns about adverse reactions, such as severe hypoglycemic events and weight gain, as well as the impact of an onerous treatment regimen on quality of life (Hermanns et al., 2010; Polonsky et al., 2011). During insulin therapy, it is still possible that blood glucose will not be within the standard range. In clinical practice, if blood glucose is not within the standard range, then the dose of insulin is increased, or the type of insulin is changed to manage blood glucose levels (Inzucchi et al., 2015). However, according to numerous previous evidence-based medical studies, hypoglycemia and weight gain are more likely to occur due to the increase in insulin during intensive treatment (Reznik and Cohen, 2013, Ukpds, 1998). In addition, the increasing number of injections will have a significant impact on treatment compliance of the patients. New therapies with improved safety and convenience are needed to address this problem. Some progress has been made to address this problem, such as the development and use of IDegLira (Morales and Merker, 2015).

IDegLira (brand name Xultophy) is a fixed ratio innovation of basal insulin [Degludec ([IDeg)] and GLP-1 RA analog [Liraglutide (Lira)] and is administered as a once-daily subcutaneous injection for the treatment of T2D. IDegLira could reduce the patients’ burden from multiple injections to a single injection every day (Hughes, 2016), and its efficacy is not affected by daily diet. Several randomized clinical trials (RCTs) (Buse et al., 2014a; Gough et al., 2014; Linjawi et al., 2017; Hirotaka et al., 2019; Kaku et al., 2019) have been conducted to investigate the efficacy and safety of IDegLira compared with its components in the treatment of T2D poorly controlled by OADs. The results showed that IDegLira has a higher glycemic benefit based on the complementary mechanism of IDeg and Lira. As documented in these RCTs, these advantages highlight the great potential of IDegLira for improving treatment adherence (Tran, 2017; Vedtofte et al., 2017).

Currently, IDeglira received regulatory approval in Europe in September 2014 and in the US in November 2016, and its cost-utility has been evaluated in the UK, Sweden, Spain, and the US(Davies et al., 2016; Ericsson and Lundqvist, 2017; Hunt et al., 2017; Raya et al., 2019). Regarding clinical outcomes, several clinical trials show evidence that IDegLira has better performance in improving the management of diabetes compared with monotherapy using IDeg or Lira. For economic outcomes, the cost and effectiveness of IDegLira in the treatment of T2D patients in other countries have been published in some studies. Barnaby Hunt et al. found that IDegLira was associated with an ICER of $63,678/QALY compared with glargine U100, indicating that IDegLira is a better strategy from a healthcare payer perspective in the US setting (Hunt et al., 2017). Marek Psota et al. (2017) found that IDegLira provides a simpler and more cost-effective option in Slovakia than basal-bolus projects. Pedro Mezquita Raya et al. (2019) showed that IDegLira has a high probability of being pharmacoeconomic versus other injectable medications for T2D patients based on real-world evidence in Spain.

In China, IDeglira was introduced in April 2022. However, there is little economic evidence in the investigation of the fixed ratio combination of IDegLira and its monotherapy of IDeg or Lira in China. This research is the first to show an estimate of the long-term economic value of IDegLira, IDeg, and Lira from a Chinese national healthcare system perspective. If IDegLira appears to not be cost-effective compared with the current price of IDeg or Lira, then a binary search is conducted to determine the best price for IDegLira and to provide reasonable suggestions to policymakers and decision-makers.

## Materials and methods

### Model overview

A cost-utility analysis based on data from six RCTs was conducted using the UK Prospective Diabetes Study Outcome Model version 2 (UKPDS OM2) (Hayes et al., 2013). UKPDS OM2 is a nonproduct-specific computer simulation model that has been validated (Keng et al., 2022) and widely used to extrapolate long-term health and economic outcomes of T2D patients from data from short-term clinical trials. The detail model structure was shown in Supplementary Figure S1. The equations in UKPDS OM2 were derived from the UKPDS 82 trial (Hayes et al., 2013), and they can be used to estimate the occurrence and survival probability of eight diabetes-related complications, such as blindness, congestive heart failure (CHF), diabetic ulcer, amputation, myocardial infarction (MI), stroke, ischaemic heart disease (IHD), and renal failure, across the lifespan of patients with diabetes. Total costs, life years and quality-adjusted life years (QALYs) were simulated by inputting data on the individual characteristics (e.g., age, duration of diabetes, and HbA1c…), risk factors (e.g., smoking history, systolic blood pressure [SBP], and history of cardiovascular events…), clinical efficacy (changes in HbA1c, bodyweight, and SBP…) and other associated parameters (e.g., time horizon, treatment time, costs, and utility…) into the UKPDS OM2. For the base case analysis (BCA) and one-way sensitivity analysis (OSA), 1000 iterations of individual cohorts were run, each with 1000 simulated patients with a default annual circle. Probabilistic sensitivity analysis (PSA) was run by Monte Carlo simulations by sampling the influencing factors with a default normal distribution. In BCA, the total simulation time (time horizon) was set at 40 years to describe the whole life years of T2D patients. The discount rate for future costs and utility was set at 5%, which aligned with WHO guidelines (Hou et al., 2019).

### Clinical data source

Baseline cohort characteristics and clinical efficacy data were sourced from six RCTs. The specific screening process and bias risk assessments can be found in the Supplementary file 2. Five RCTs [NCT02607306 (Kaku et al., 2019), NCT01392573 (Buse et al., 2014b), NCT01336023 (Gough et al., 2014), NCT03175120 (Pei et al., 2021), NCT02911948 (Watada et al., 2019)] were published, and the results of one of the RCTs [NCT03172494 (2017)] were uploaded into https://www.clinicaltrials.gov. Detailed information about the six RCTs is shown in the Supplementary Material S2. The synthesized cohort baseline characteristics that were input into the model are shown in Table 1. In our patient cohort, each treatment group (the IDegLira group, the IDeg group, and the Lira group) was simulated as 1000 patients with clinically diagnosed T2D. In the IDegLira group, patients received IDegLira (100 U/3.6 mg per ml) once daily (OD) subcutaneously. In the IDeg group, patients received insulin degludec (IDeg: 100 U/ml) OD subcutaneously. In the Lira group, patients received liraglutide (6 mg/ml) OD subcutaneously. In the cohort, the mean age [standard deviation (SD)], duration of diabetes, initial HbA1c, and mean BMI were 55.6 (10) years, 8.87 (5.92) years, 8.84 (0.94) %, 29.68 (4.79) kg/m2, respectively. The efficacy data are displayed in Table 2. In the short-term RCTs, the overall treatment effects on HbA1c at 26 2weeks were −1.94%, −1.29%, and −1.42% for IDegLira, IDeg, and Lira, respectively. The effects on body weight were −0.18, 1.65, and −2.26 kg for IDegLira, IDeg, and Lira, respectively. Changes in SBP from the endpoint to baseline were −1.78, 1.09, and −2.33 mmHg in the three groups. All interventions were assumed to last for 5 years to simulate the clinical practice and to align with the previous cost-utility analysis of IDegLira (Raya et al., 2019).

**TABLE 1:** Baseline characteristics of input cohort patients synthesized from 6 RCTs.

Trial characteristics	Overall baseline	
	Mean (%)	SD
Total simulation sample	1000	—
Mean age, years	55.60	10.00
Female, %	41.88%	—
Race, %		
White	31.39%	—
Black/African American	3.47%	—
Asian	64.12%	—
Other*	1.02%	—
Duration of diabetes, years	8.87	5.92
Mean HbA1c, %	8.44	0.94
BMI, kg/m^2	29.68	4.79
Mean body weight, kg	82.33	16.92
Height, meters	1.67	1.88
SBP, mmHg	133.20	12.76

Note: RCT, randomized clinical trial; BMI, body mass index; SBP, systolic blood pressure; SD, standard deviation.

**TABLE 2:** The main efficacy data for three treatment arms synthesized from 6 RCTs.

Outcomes	Overall baseline		IDegLira		IDeg		Lira	
	Mean	SD	Mean	SE	Mean	SE	Mean	SE
Mean HbA1c, %	8.44	0.94	−1.94	0.02	−1.29	0.03	−1.42	0.03
Mean bodyweight, kg	82.33	16.92	−0.18	0.07	1.65	0.10	−2.26	0.12
Mean SBP, mm Hg	133.20	12.76	−1.78	0.41	1.09	0.51	−2.33	0.65

Note: IDegLira, fixed ratio combination of insulin degludec and liraglutide; IDeg, insulin degludec; Lira, Liraglutide; SE, standard error.

### Costs and utility

Costs were based on a Chinese national healthcare system perspective and were expressed in 2021 US dollars. Medication costs were calculated based on wholesale pack costs, mean daily doses and injection frequency. The wholesale costs per package of IDegLira, IDeg and Lira were sourced from the average official bid price in China in 2021. The mean daily doses were assumed to be the average of the maximum daily doses among the six RCTs. The injection frequency of the three drugs was once daily. According to new insulin delivery recommendations (Frid et al., 2016), IDegLira, IDeg, and Lira were assumed to be injected by individually packaged injection needles. In each group, patients were assumed to use 1 self-monitoring of blood glucose (SMBG) test per day. Direct costs of diabetes, such as annual medication costs (IDegLira, IDeg and Lira), needles, SMBG testing, and costs associated with the presence or absence of diabetes-related complications, were determined in this study (Tables 3, 4). The cost in the absence of complications was sourced from a cohort study performed in China (Li et al., 2019). The diabetes-related complication costs were extracted from previous economic studies (Shao et al., 2017; Wu et al., 2018a; Wu et al., 2018b; Cai et al., 2019) in a Chinese setting and inflated to 2021 US dollars using the consumer price index (CPI). Health state utility scores ranged from 0 to 1 were used to assess health-related quality of life (HRQoL). HRQoL associated with T2D with or without complications was derived from published literature (Clarke et al., 2002; Pan et al., 2016; Shao et al., 2017; Wu et al., 2018a; Wu et al., 2018b; Cai et al., 2019). The 0.876 of initial utility for T2D patients without complications was sourced from a 5-level, 5-dimensional EuroQol scale (EQ–5D–5L) study focusing on Chinese T2D patients. The utility decrements for each diabetes complications were derived from published literature on Chinese T2D patients, UKPDS 62 study, and other studies (Clarke et al., 2002; Pan et al., 2016; Shao et al., 2017; Wu et al., 2018a; Wu et al., 2018b; Cai et al., 2019). The detailed data of costs and utilities are shown in Table 4.

**TABLE 3:** Costs of medications.

Drug name	Dosage form	Specification	Unit of cost (¥/box)	Usage	Mean daily doses^a^	Annual cost ($)
IDegLira	Injection	3 ml: 100 U IDeg and 3.6 mg/ml Lira	499	OD subcutaneously	34.62 U	3257.91
Ideg	Injection	3 ml*300 U*1 piece/box	79.2	OD subcutaneously	40.33 U	602.37
Lira	Injection	3 ml*18 mg*1 piece/box	339	OD subcutaneously	1.8 mg	1917.93

Note: OD, once daily.

^a^ Mean daily doses were sourced from the average maximin daily dose of 6 RCTs. 1$ = ¥6.4515 (2021).

**TABLE 4:** Costs and utilities of diabetes-related complications.

Model inputs	At time of event			In subsequent years	
	Fatal cost	Non-fatal cost	Utility decrement	Cost	Utility decrement
IHD	0	6959.65	−0.09	1242.47	−0.09
MI	8686.87	8686.87	−0.055	535.83	−0.236
Heart failure	3354.95	3354.95	−0.236	1773.97	−0.236
Stroke	2506.28	3382.97	−0.164	596.42	−0.326
Amputation	4904.14	4904.14	−0.38	4773.72	−0.38
Blindness	—	2611.53	−0.157	1931.98	−0.157
Renal failure	0	16240.90	−0.4	16240.90	−0.4
Ulcer	—	2554.59	−0.059	899.09	−0.059
Initial utility	0.876				
Diabetes management cost^a^	1717.45				

Note: IHD, ischemic heart disease; MI, myocardial infarction.

^a^ Diabetes management cost contains cost of needles, cost of SMBG testing, and cost in the absence of complications.

### Cost-utility analysis

The UKPDS OM2 model was applied to simulate the progression, health outcomes and relevant costs of T2D over a 40-year period, with a default cycle length of 1 year. The study showed a comparison of IDegLira, a novel fixed-ratio combination product, with its individual therapies, IDeg or Lira, as the comparators in the treatment of T2D patients. Incremental cost-utility ratios (ICUR) and incremental net monetary benefits (INMB) were adopted as results interpretation indicators. The calculation formula for ICUR was the ratio of incremental differences in costs (ΔCosts) and QALYs (ΔQALYs) between the intervention and the comparators. The formula for INMB was “ΔQALYs multiplied by the willingness-to-pay (WTP) threshold and then subtracting ΔCosts”. The WTP threshold of 1–3 times the gross domestic product (GDP) per capita as recommended in the WHO guidelines for developing countries was used to determine the pharmacoeconomic of the drugs, which was predefined as $37654.50/QALYs in this study. A positive value of INMB and a value of ICUR that surpasses the WTP threshold indicate that the intervention is cost-effective.

### Uncertainty analyses

Uncertainty analyses of OSA and PSA for all three groups were performed to evaluate the impacts of parameter uncertainty on the BCA results. The OSA was conducted to separately examine the robustness and generalizability of parameters including costs, utilities, discount rate, time horizon, and treatment time. Costs of complications and health disutility scores varied between their 95% confidence intervals (95% CIs). If their 95% CIs were not reported, costs and utility scores were adjusted by ± 20% and ±10%, respectively, such as IHD and ulcer. The detailed variations were shown in Table 5. The OSA results were expressed as a tornado diagram. PSA was run with a normal distribution for influencing factors, and its results are depicted as scatter plots. Scenario analyses were applied by binary search to search the extent of price reduction for IDegLira if it was too expensive for patients.

**TABLE 5:** Parameters of sensitivity analysis.

Parameters	Baseline	Low	High
Discount rate	5%	3%	8%
Initial utility	0.876	0.78	0.92
Treatment time	5	4	6
Time horizon	40	30	50
^a^Cost, $			
Needle per year cost	139.25	100.14	181.61
^b^IHD per year cost (±20%)	1242.47	993.98	1490.96
MI per year cost	535.82	339.57	732.08
Heart failure per year cost	1773.97	1476.17	3097.18
Stroke per year cost	596.42	524.65	974.23
Blindness per year cost	1931.99	1683.01	2180.83
Renal failure per year cost	16240.89	15476.81	17142.17
Amputation per year cost	4773.70	0.00	8481.37
^b^Ulcer per year cost (±20%)	899.09	719.27	1078.91
^a^Health disutility scores			
^b^IHD disutility scores (±10%)	0.09	0.081	0.099
MI disutility scores	0.236	0.026	0.446
Heart failure disutility scores	0.236	0.026	0.446
Stroke disutility scores	0.326	0.036	0.616
Blindness disutility scores	0.157	0.007	0.307
Renal failure disutility scores	0.4	0.19	0.61
Amputation disutility scores	0.38	0.204	0.496
^b^Ulcer disutility scores (±10%)	0.059	0.0531	0.0649

^a^ Costs of complications and health disutility scores varied between their 95% CIs.

^b^ Costs and utility scores of IHD and ulcer were adjusted by ± 20% and ±10%, respectively, as their 95% CIs were not reported.

Probabilistic sensitivity analysis was run by Monte Carlo simulations by sampling the influencing factors with a default normal distribution.

## Results

After a life year (40 years) simulation, the BCA results are shown in Table 6 in 2021 US dollars. Regarding health outcomes, T2D patients who were uncontrolled on an OAD and received IDegLira, IDeg and Lira gained 11.79 QALYs, 11.62 QALYs, and 11.73 QALYs, respectively. Regarding medication costs, IDegLira, IDeg, and Lira cost $20281.61, $3726.76, and $11941.26, respectively. Regarding complication costs, IDegLira, IDeg, and Lira cost $25274.22, 25016.67, and $25204.84, respectively. Regarding total costs, IDegLira, IDeg, and Lira cost $45555.83, $28743.43, and $36660.18, respectively. Among the three treatment groups, IDegLira resulted in the highest QALYs and the highest costs, simultaneously.

**TABLE 6:** Long-term cost-utility outcomes.

Mean (95% CIs)	IDegLira	Comparator	Difference
IDegLira vs. Ideg			
LEs, years	13.96 (13.75, 14.17)	13.76 (13.56, 13.99)	0.19
QALYs, years	11.79 (11.63, 11.99)	11.62 (11.46, 11.83)	0.17
Therapy costs, $	20281.61 (19679.76, 20708.26)	3726.76 (3615.76, 3814.06)	16554.85
Complication costs, $	25274.22 (24892.50, 25626.36)	25016.67 (24637.76, 25404.47)	257.55
Total costs, $	45555.83 (44689.68, 46169.20)	28743.43 (28300.94, 29166.47)	16812.4
ICUR	—	—	$99464.12 per QALY
Absolute NMB	398496.76	408944.42	
INMB	—	—	−10447.67
IDegLira vs. Lira			
LEs, years	13.96 (13.75, 14.17)	13.90 (13.72, 14.13)	0.06
QALYs, years	11.79 (11.63, 11.99)	11.73 (11.59, 11.95)	0.06
Therapy costs, $	20281.61 (19679.76, 20708.26)	11941.26 (11651.43, 12249.66)	8340.35
Complication costs, $	25274.22 (24892.50, 25626.36)	25204.84 (24906.88, 25646.46)	69.38
Total costs, $	45555.83 (44689.68, 46169.20)	37146.10 (36660.18, 37774.14)	8409.73
ICUR	—	—	$143348.26 per QALY
Absolute NMB	398496.76	404697.43	
INMB	—	—	−6200.68

Note: 95% CIs, 95% confidence intervals; LEs, life expectancy; QALYs, quality-adjusted life years; ICUR, incremental cost-utility ratio; Absolute NMB, absolute net monetary benefit; INMB, incremental net monetary benefit. WTP threshold: $37654.50/QALYs.

Compared with IDeg, IDegLira had an ICUR value of $99464.12/QALYs, which was surpassed the WTP threshold, indicating that IDegLira was not a cost-effective strategy. Compared with Lira, IDegLira, with an ICUR of $143348.26/QALYs that surpassed the WTP threshold, was not an economic strategy. The INMBs of IDegLira versus IDeg and IDegLira versus Lira were −10447.67 and −6200.68, respectively, demonstrating the same results as ICURs. The order of absolute NMB was IDeg, Lira, and IDegLira, further confirming the base case conclusion. In the base case analysis, IDegLira was not a cost-effective therapy because of high QALYs and high total costs compared with those of IDeg and Lira.

**FIGURE 1:**
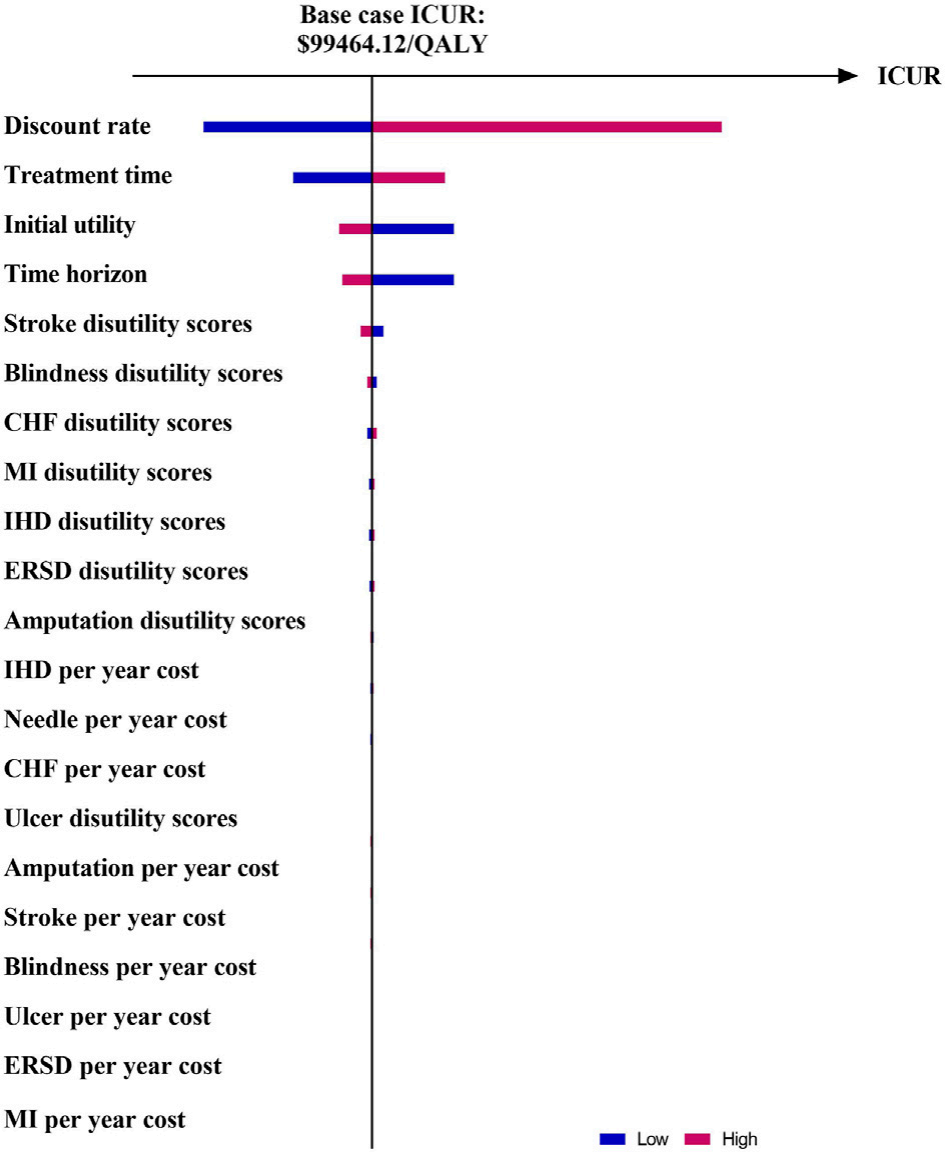
Tornado diagram of ICUR in IDegLira vs. IDeg. Figure note: WTP threshold: $37654.50/QALYs.

**FIGURE 2:**
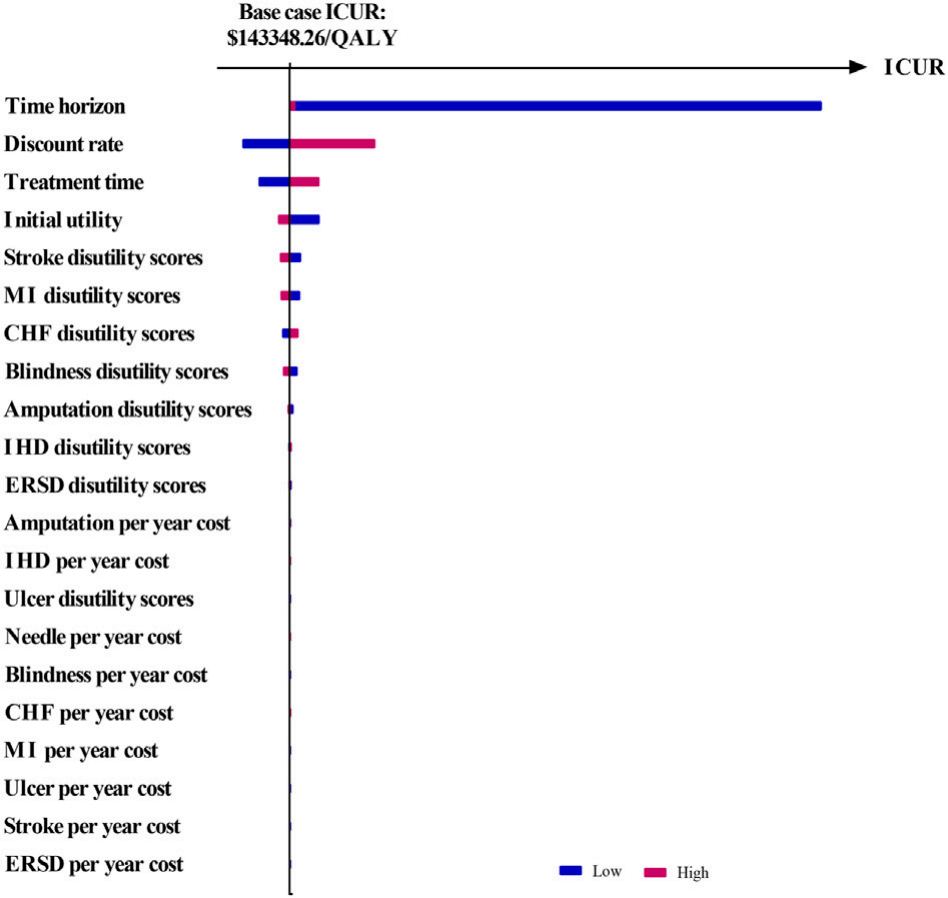
Tornado diagram of ICUR in IDegLira vs. Lira. Figure note: WTP threshold: $37654.50/QALYs.

**FIGURE 3:**
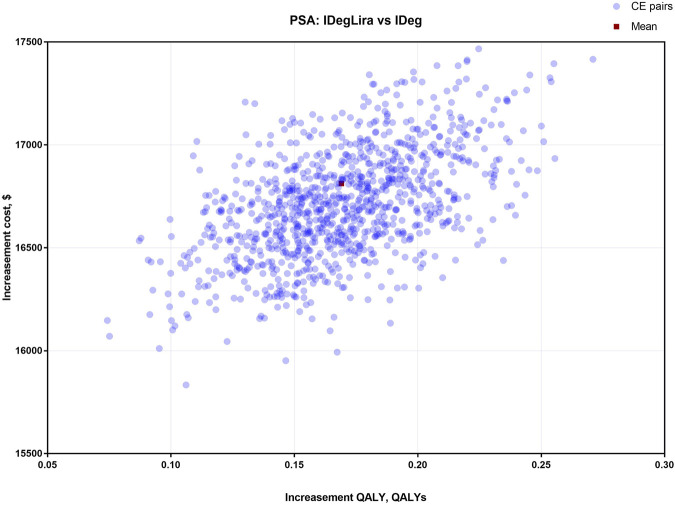
Scatter plots of ICUR for the treatment with IDegLira vs. IDeg.

**FIGURE 4:**
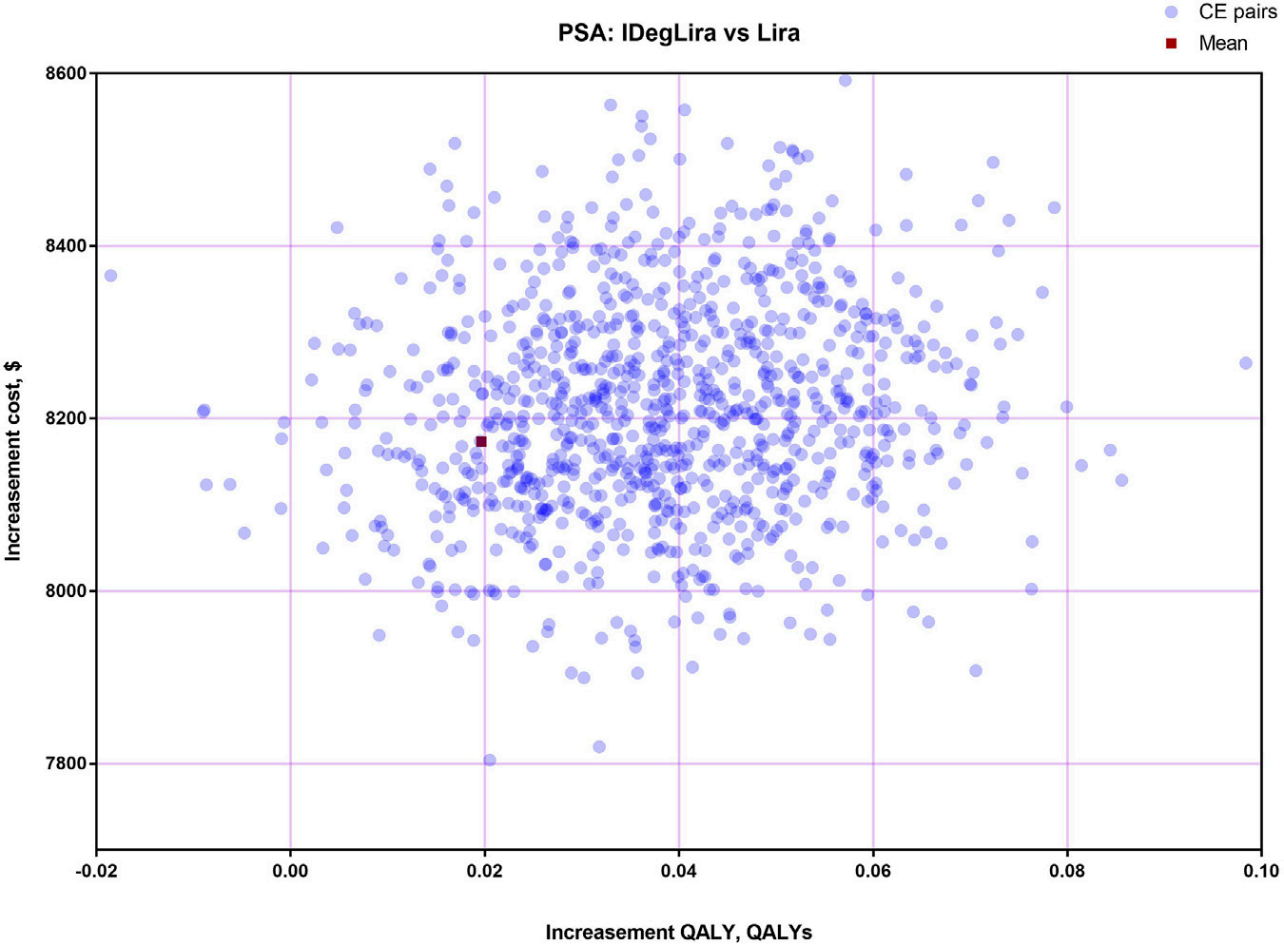
Scatter plots of ICUR for the treatment with IDegLira vs. Lira.

The results of OSA are expressed as a tornado diagram and shown in Figures 1, 2. In Figure 1, the discount rate, treatment time, initial utility, and time horizon had a more substantial impact on the base case results than the other influencing factors. In Figure 2, the ICUR of IDegLira versus Lira was more sensitive to the variations in the time horizon, discount rate, treatment time, and initial utility. However, all the ICURs were under the WTP threshold and did not reverse the BCA conclusion. The OSA results indicate that the base case conclusion was reliable for different simulation scenarios and that the current price of IDegLira is still not cost-effective compared with that of IDeg and Lira. Figures 3, 4 present the scatter plots of ICURs resulting from PSA performed in the model. In Figure 3, all iterations are located above the WTP threshold, revealing that there is little chance for IDegLira to be cost-effective compared with IDeg in the treatment of T2D patients who were uncontrolled by OADs. Figure 4 shows that there was no chance of IDegLira being cost-effective compared with Lira. There was a 0.9% probability that IDegLira was associated with lower QALYs and higher costs. Compared with Lira, IDegLira had a 0% chance of being cost-effective and a 0.9% chance of being undominant. In conclusion, the results of BCA, OSA, and PSA show that the current price of IDegLira is not cost-effective. Therefore, it is imperative to reduce the price of IDegLira by adding medicine to the National Medical Insurance System in China. Hence, scenario analyses were applied by reducing the price of IDegLira. Some assumptions were tested using a binary search to investigate the appropriate price reduction for IDegLira, taking the cost of IDeg or Lira as a comparator. Detailed assumptions and results are shown in Supplementary Material S3. The ICUR of $37654.50/QALYs, which approached the WTP threshold, was achieved when the annual cost of IdegLira was $1375.23, indicating that, at the price, IDegLira would become equally as cost effective as IDeg. IDegLira would be equally as cost-effective as Lira when the annual cost of IDegLira was set at $2262.88. Therefore, our study found that IDegLira appears to only be cost-effective if the annual cost of IDegLira is decreased by 58% when considering IDeg as a reference, or by 30.57% when considering Lira as a reference.

## Discussion

The recommendation by the American and European Association for T2D patients emphasizes that the cornerstone of success for T2D treatment is based on the consideration of the needs, preferences, and tolerance of each patient, and more patient-centered individualized therapeutic strategies are needed (Inzucchi et al., 2012). Because of the progression of T2D and the degradation of β-cell function, most T2D patients ultimately need an intensive treatment plan to maintain glycaemic targets (Davies et al., 2018). For T2D patients who are not sensitive to OADs and basal insulin medication, an intensive strategy with IDegLira could be suggested as a further treatment to achieve individual glycaemic goals. Currently, very few fixed-ratio soluble combinations of insulin and GLP-1 RA drugs have been approved by the Food and Drug Administration. IDegLira is a novel combination of insulin degludec and GLP-1 RA liraglutide and is associated with better glycemic and bodyweight benefit.

The complementary mechanisms of a basal insulin and a GLP-1 RA allow IDegLira to achieve glycemic control and manage body weight when compared to other therapy strategies for patients with uncontrolled T2D on basal insulin, such as increasing doses of basal insulin. People who receive basal insulin may suffer from injection pain caused by approximately four daily injections as part of a basal-bolus regimen and two daily injections of Lira added to basal insulin (Hunt et al., 2017). IDegLira is packed in a single-injection pen of a fixed-ratio of IDeg and Lira, which means that patients using IDegLira do not need multiple daily injections. A reduce injection burden may be attractive to T2D patients because a single device of IDegLira is packed with a combination of IDeg and Lira, which is titrated simultaneously (Boye et al., 2011). Furthermore, the US FDA guidelines describe that any new hypoglycemic drugs should not lead to an undesirable impact on the cardiovascular system (US Department of Health and Human Services, 2008). Lira can reduce the formation of atherosclerotic plaques. Therefore, IDegLira has a general benefit in reducing cardiovascular risk markers, considering basal insulin or basal–bolus therapy as a comparator (Vilsbøll et al., 2019).

In our cost-utility analysis, the ICURs of IDegLira versus IDeg and IDegLira versus Lira are $99464.12/QALYs and $143348.26/QALYs, respectively, which both surpassed the WTP threshold. Although IDegLira achieved the highest QALYs among the three, it also has the highest expenditure as it is new to the Chinese market. We found that IDegLira, at the present price, appears to not be cost-effective when compared with its monotherapy. The BCA results are robust to OSA and PSA. Therefore, the market price of IDegLira may be further priced through government negotiation or volume-based procurement in the future. Our study found that IDegLira appears to only be cost-effective if the annual cost of IDegLira is decreased by 58% when considering IDeg as a reference or by 30.57% when considering Lira as a reference.

Some potential limitations of this study are discussed as follows. First, due to the lack of long-term clinical evidence for the use of IDegLira, IDeg, and Lira in the treatment of T2D patients, short-term RCT data were applied to predict lifetime health outcomes. Second, long-term model simulations rely on model equations derived from the UKPDS 82 trial (Hayes et al., 2013) that is mainly based on White Caucasian, Afro-Caribbean and Asian-Indian populations. Therefore, when the model results are extrapolated to the Chinese population, decision-makers should take this conclusion seriously and with caution. Third, most diabetes complication costs, and disutility were sourced from Chinese population studies, while some were extracted from the UKPDS 62 study, which was mainly based on the White population. This may cause some bias in the BCA results. To make the conclusion more credible, sensitivity analyses were performed to solve the uncertainty of input parameters, such as population bias, utility bias and model bias. Last, from a Chinese national healthcare system perspective, only direct costs were considered. This means that hidden costs, including delay in compensation, nonmedical costs, and costs of nursing management costs, may influence the results in real-world scenarios. In real-world clinical practice, multidimensional factors should be considered with economic suggestions as an extra reference.

## Conclusion

In conclusion, from a Chinese national healthcare system perspective, IDegLira appears to not be cost-effective when compared with IDeg or Lira. However, after a binary search, IDegLira appears to only be cost-effective if the annual cost of IDegLira is decreased by 58% when considering IDeg as a reference or by 30.57% when considering Lira as a reference.

## Data Availability

The original contributions presented in the study are included in the article/Supplementary Material, further inquiries can be directed to the corresponding authors.

## Abbreviations

T2D, type 2 diabetes; QALYs, quality-adjusted life years; RCTs, randomized clinical trials; OSA, one-way sensitivity analysis; IDegLira, fixed ratio combination of insulin degludec and liraglutide; IDeg, insulin degludec; Lira, liraglutide; PSA, probabilistic sensitivity analysis; ICUR, incremental cost-utility ratio; IDF, International Diabetes Federation; OADs, oral hypoglycemic drugs; UKPDS OM2, UK Prospective Diabetes Study Outcome Model version 2; CHF, congestive heart failure; MI, myocardial infarction; SBP, systolic blood pressure; BCA, base case analysis; OD, once daily; BMI, body mass index; SD, standard deviation; SE, standard error; SMBG, self-monitoring of blood glucose; CPI, consumer price index; HRQoL, health-related quality of life; INMB, incremental net monetary benefits; WTP, willingness-to-pay; GDP, gross domestic product
