# Supplementary material for: Insulin degludec/liraglutide versus its monotherapy on T2D patients: A lifetime cost-utility analysis in China
Source: Front Pharmacol. 2022 Nov 18;13:1011624. doi: 10.3389/fphar.2022.1011624 (PMC9716023; doi:10.3389/fphar.2022.1011624)
Supplement: Supplementary file 1 [file Table1.DOCX]

**The therapy of type 2 diabetes with ﻿fixed-ratio combination insulin degludec/liraglutide (IDegLira) versus its monotherapy (insulin degludec or liraglutide): A long-term cost-effectiveness analysis in the Chinese setting**

**Supplementary file 1:**

**Methods**

**1.1 Data source and search strategy**

We systemically searched PubMed、Web of science、ScienceDirect、Ovid、Embase、CNKI、clinicaltrial.gov for eligible studies from the inception to November 30, 2021. We used the combination of the following medical subject heading (MeSH) and free-text terms: type 2 diabetes, IdegLira. In addition, we searched clinicaltrials.gov to identify clinical trials and checked for data availability of any relevant trials to make sure that no research is missed. The detailed search strategy was clearly described in **Table S1**.

**Table S1** The retrieval strategies of each database

| The database | Retrieval strategy formula |
| --- | --- |
| PubMed | 1."Ideglira"[Title/Abstract]  2. "type 2 diabetes"[Title/Abstract]  3."Ideglira"[Title/Abstract] AND "type 2 diabetes"[Title/Abstract] |
| Web of Science | (TS=(Type 2 Diabetes)) AND TS=( Ideglira) |
| ScienceDirect | Type 2 Diabetes AND Ideglira |
| Ovid | (Ideglira and Type 2 Diabetes).ab. |
| Embase | 1.'ideglira:ab,ti AND 'type 2 diabetes':ab,ti  2.'#1 AND ('randomized controlled trial'/de OR 'randomized controlled trial topic'/de) |
| CNKI | （Subject：Type 2 Diabetes（Accurate））AND（Subject：Ideglira（Accurate）） |

**1.2 Study selection**

Two researchers independently selected eligible studies which were included in the meta-analysis. If there existed a disagreement, they would solve through communication and discussion. If no consensus can be reached, they would solve by consulting another researcher.

**Inclusion criteria** were listed as the followings: (1) The study subjects were patients with type 2 diabetes who were at least 18 years old when they entered the clinical trial; (2) The duration of diabetes should be at least 6 months; (3) For diabetics with poor blood glucose control: HbA1c (glycosylated hemoglobin) is 7.0-11.0% (including both); (4) BMI is greater than or equal to 20 kg/m^2; (5) after at least one oral hypoglycemic agent (OAD) treatment for at least 60 days prior to the trial; (6) The study type was randomized controlled clinical trial (RCT); (7) The study protocol focused on the difference in clinical efficacy and safety between IDegLira and IDge or Lira; (8) The main outcome indicators were ΔHbA1c; (9) Follow-up was 12 weeks or longer; (10) Research results and data are publicly available.

**Studies were excluded** if they are: (1) The study protocol did not focus on the difference in clinical efficacy and safety between IDegLira and IDge or Lira; (2) not for type 2 diabetes; (3) Impaired liver and kidney function; (4) Studies based on cells or animals; (5) Other types of studies except RCT; (6) Study of single drug design.

**1.3 Data extraction**

Two researchers working independently abstracted data from eligible studies using a predesigned extraction form, and any disagreements were arbitrated by discussing with a third researcher. Data collected include but are not limited to first author, publication date, literature type, sample size, intervention, study duration, outcome indicators.

**1.4 Quality assessment**

Two researchers assessed the risk of bias in the eligible studies with the Cochrane Collaboration’s tool. The risk of bias was described and assessed in seven specific domains: random sequence generation, allocation concealment, blinding of participants and personnel, blinding of outcome assessment, incomplete outcome data, selective reporting and other bias. The results of these domains were graded as a ‘low’ risk of bias, a ‘high’ risk of bias or an ‘unclear’ risk of bias, and any disagreements were resolved by consensus.

**1.5 Data synthesis and statistical analysis**

All analyses were performed using Review Manager5.3 software provided on the Cochrane official website. Heterogeneity between studies was assessed using Cochrane Q statistics and I^2^ statistics. According to the results of heterogeneity test, different models were selected to combine effect sizes. Statistical heterogeneity was assessed with the I^2^ statistic considering values below 50% indicative of low heterogeneity， multiple similar studies can be considered to be homogenous, and Fixed effects model is adopted to merge statistics. When the heterogeneity is too high (I^2^≥50%), or the heterogeneity still does not decrease significantly after sensitivity analysis, the results of multiple studies are considered to be heterogeneous, and the Random effects model is used to merge the statistics. Relative ratio（RR）and 95% confidence intervals (CI)were applied to dichotomous outcomes, whereas Mean different（MD）and 95% CI were applied to continuous outcomes. Two-tailed, p < 0.05 was considered statistically significant.

**Results**

**2.1 Results of Search**

This study searched PubMed, Web of Science, ScienceDirect, Ovid, Embase and CNKI databases according to the retrieval strategy, and found a total of 585 relevant literatures. After literature screening according to the set inclusion criteria, 6 RCTS were eventually included, 5 of which published literatures consistent with this study, and one of which was obtained by searching the clinical trial database Clinicaltrials.gov, but this RCTS did not publish relevant literatures consistent with this study. Results of the literature selection process are depicted in **Figure S1**. The detail data of the 6 RCTs are shown in **Table S2**.

**
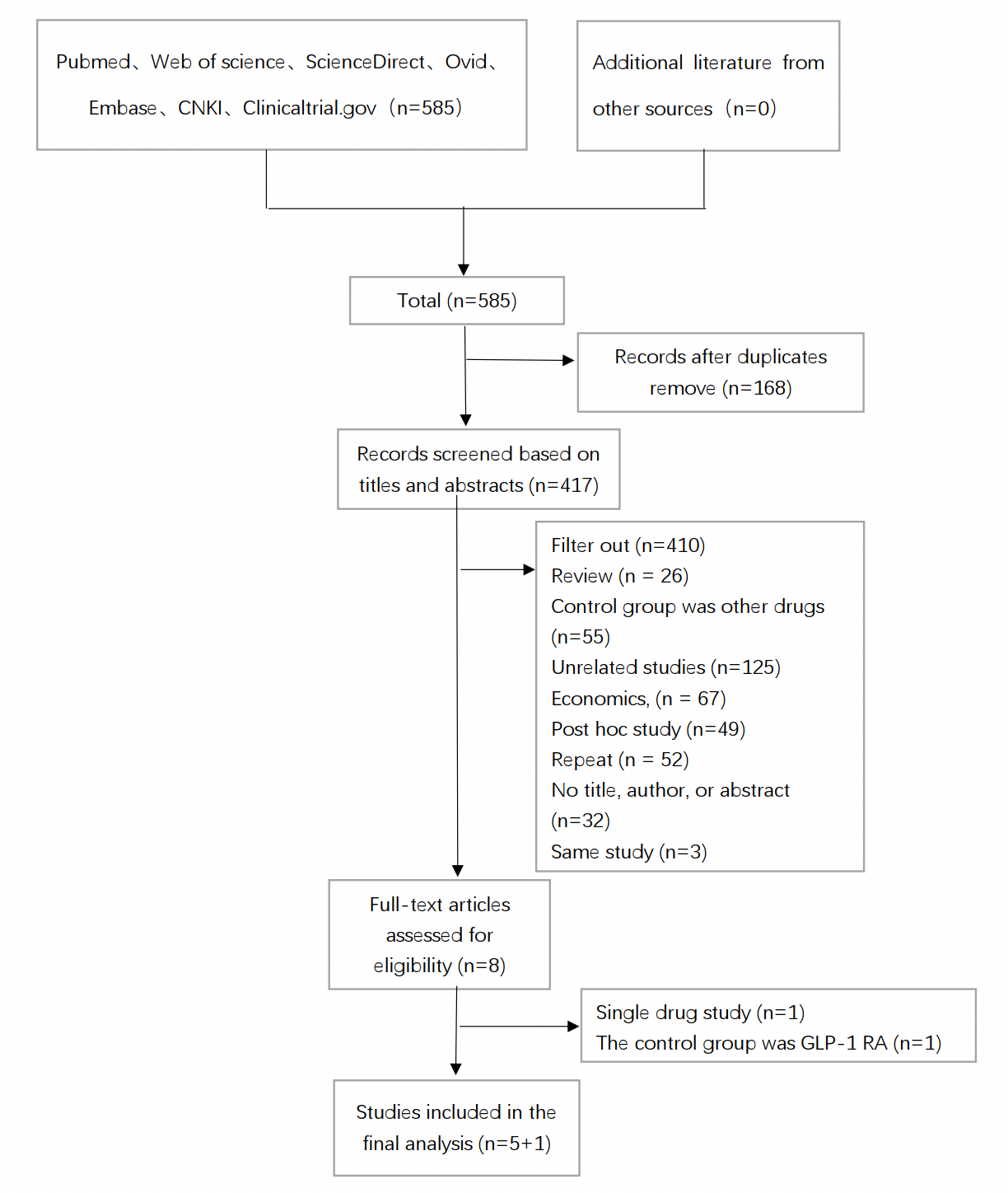
**

**Figure S1** Flow chart of literature screening.

**Table S2** The detail data of 6 enrolled RCTs.

| NO. of clinical trials | Study | Year | Country | Duration of trial | Intervention | No. of patients | Age (SD) | BMI (SD) | Initial HbA1c (SD) |
| --- | --- | --- | --- | --- | --- | --- | --- | --- | --- |
| NCT02607306 | Kohei Kaku | 2019 | Japan | 52w | IdegLira | 275 | 56.9(10.2) | 26.1(3.7) | 8.52(1.12) |
|  |  |  |  |  | Ideg | 271 | 57.8(9.9) | 26.6(4.8) | 8.53(1.05) |
|  |  |  |  |  | Lira | 273 | 56.8(10.1) | 26.5(4.5) | 8.32(0.99) |
| NCT01392573 | JohnB.Buse | 2014 |  | 26w | IdegLira | 199 | 56.8(8.9) | 33.6(6) | 8.7(0.7) |
|  |  |  |  |  | Ideg | 199 | 57.5(10.5) | 33.8(6) | 8.8(0.7) |
| NCT01336023 | Gough, S. C. | 2014 |  | 26w | IdegLira | 833 | 55.1(9.9) | 31.2(5.2) | 8.3(0.9) |
|  |  |  |  |  | Ideg | 413 | 54.9(9.7) | 31.2(5.3) | 8.3(1) |
|  |  |  |  |  | Lira | 414 | 55(10.2) | 31.3(4.8) | 8.3(0.9) |
| NCT03175120 | YuPei | 2021 | China | 26w | IdegLira | 289 | 54.5(9.8) | 27.5(3.3) | 8.93(1.2) |
|  |  |  |  |  | Ideg | 138 | 55.3(10) | 27(2.9) | 8.96(1.17) |
| NCT03172494 |  | 2017 | China | 26w | IdegLira | 361 | 54.5(10.3) |  | 8.2(0.83) |
|  |  |  |  |  | Ideg | 179 | 55.7(10.2) |  | 8.31(0.84) |
|  |  |  |  |  | Lira | 180 | 54.1(10.2) |  | 8.21(0.77) |
| NCT02911948 | Hirotaka Watada | 2019 | Japan | 26w | IdegLira | 105 | 56.6(10.4) | 27.3(3.1) | 8.61(0.88) |
|  |  |  |  |  | Ideg | 105 | 55.5(10) | 28.1(4.4) | 8.64(2.52) |

**2.2 Risk of Bias Assessment**

Risk of bias assessment is presented in **Figure S2** and **Figure S3**. A total of 6 studies explicitly described the random sequence generation, mainly by interactive voice response system or web response system. And all studies used unpredicted methods to generate a random sequence which stated a low risk of allocation concealment process. In total, 3 studies indicated that they adopted a double-blind design, while 2 studies employed an open design. But only 2 studies have specifically described the practice of blinding. All studies provided data for our primary outcome; therefore, all studies were regarded as having a low risk in this domain. All included studies were considered to have a low risk of bias in selective reporting, according to the review of their protocols in ClinicalTrials.gov. One study was considered to have an unclear risk of bias in the domain of other bias due to no qualified literature was published.


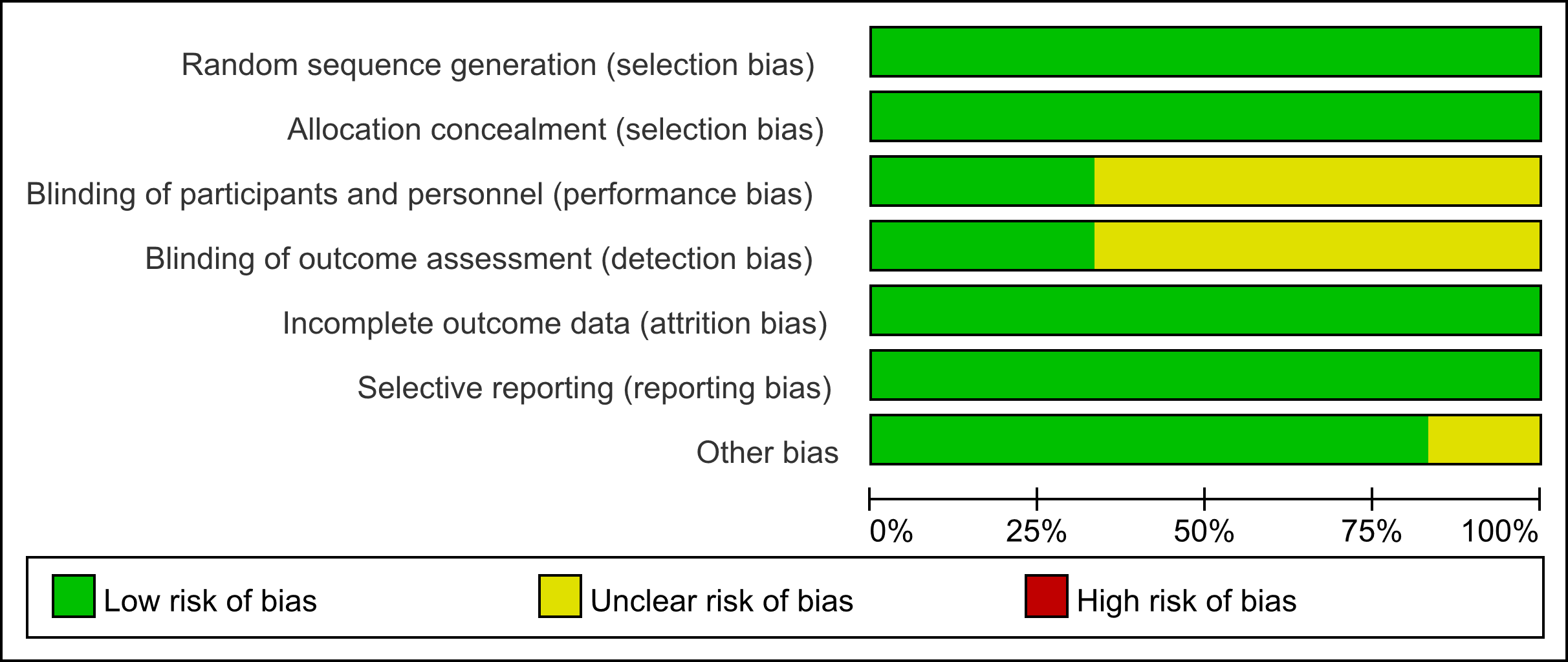


**Figure S2** Diagram of risk bias for included studies


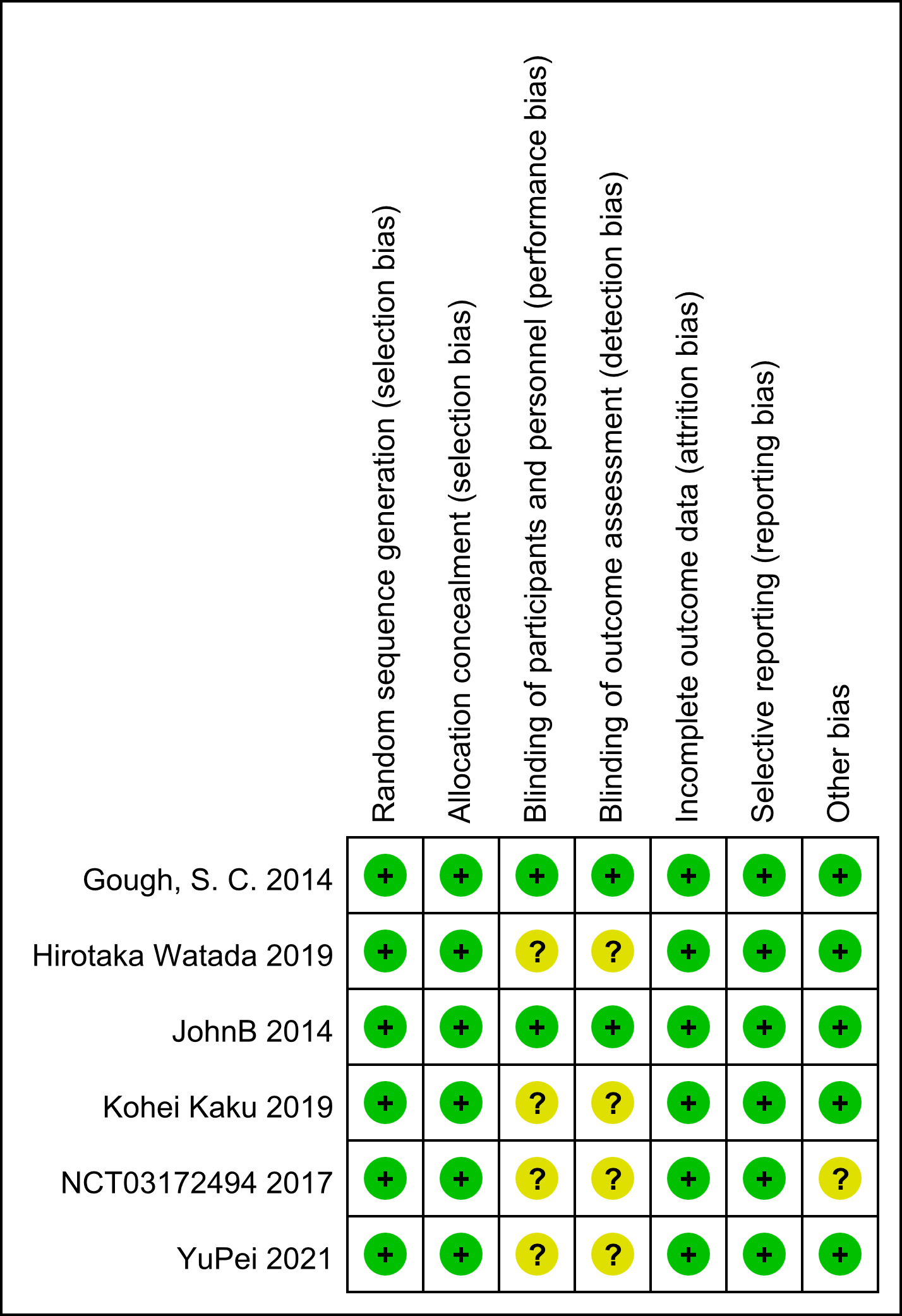


**Figure S3** Schematic diagram of risk bias for included studies

**Supplementary file 2:**

**Table S1** Outputs for binary search for further reduction in the annual cost of IDegLira, taking that of IDeg as a reference.

| NO. | Group | ∆QALY | ∆cost | ICUR | Relationship with 3 GDP |
| --- | --- | --- | --- | --- | --- |
| A | Baseline | $3,257.91 | | | |
|  | IDegLira | 0.16902981 | 16812.4015 | 99464.1185 | > |
|  | Ideg |  |  |  |  |
| B | A/2 | $1,628.95 | | | |
|  | IDegLira | 0.16902981 | 8519.72705 | 50403.6939 | > |
|  | Ideg |  |  |  |  |
| C | B/2 | $814.48 | | | |
|  | IDegLira | 0.16902981 | 1601.18645 | 9472.80484 | < |
|  | Ideg |  |  |  |  |
| D | (B+C)/2 | $1,221.71 | | | |
|  | IDegLira | 0.16902981 | 5060.45675 | 29938.2494 | < |
|  | Ideg |  |  |  |  |
| E | (B+D)/2 | $1,425.33 | | | |
|  | IDegLira | 0.16902981 | 6790.0919 | 40170.9717 | > |
|  | Ideg |  |  |  |  |
| F | (D+E)/2 | $1,323.52 | | | |
|  | IDegLira | 0.16902981 | 5925.27432 | 35054.6105 | < |
|  | Ideg |  |  |  |  |
| G | (E+F)/2 | $1,374.43 | | | |
|  | IDegLira | 0.16902981 | 6357.68311 | 37612.7911 | < |
|  | Ideg |  |  |  |  |
| H | (E+G)/2 | $1,399.88 | | | |
|  | IDegLira | 0.16902981 | 6573.8875 | 38891.8814 | > |
|  | Ideg |  |  |  |  |
| I | (G+H)/2 | $1,387.16 | | | |
|  | IDegLira | 0.16902981 | 6465.78531 | 38252.3362 | > |
|  | Ideg |  |  |  |  |
| J | (G+I)/2 | $1,380.79 | | | |
|  | IDegLira | 0.16902981 | 6411.73421 | 37932.5637 | > |
|  | Ideg |  |  |  |  |
| K | (G+J)/2 | $1,377.61 | | | |
|  | IDegLira | 0.16902981 | 6384.70866 | 37772.6774 | > |
|  | Ideg |  |  |  |  |
| L | (G+K)/2 | $1,376.02 | | | |
|  | IDegLira | 0.16902981 | 6371.19588 | 37692.7342 | > |
|  | Ideg |  |  |  |  |
| M | (G+L)/2 | $1,375.22 | | | |
|  | IDegLira | 0.16902981 | 6364.4395 | 37652.7627 | < |
|  | Ideg |  |  |  |  |
| N | (L+M)/2 | $1,375.62 | | | |
|  | IDegLira | 0.16902981 | 6367.81769 | 37672.7485 | > |
|  | Ideg |  |  |  |  |
| O | (M+N)/2 | $1,375.42 | | | |
|  | IDegLira | 0.16902981 | 6366.12859 | 37662.7556 | > |
|  | Ideg |  |  |  |  |
| P | (M+O)/2 | $1,375.32 | | | |
|  | IDegLira | 0.16902981 | 6365.28404 | 37657.7591 | > |
|  | Ideg |  |  |  |  |
| Q | (M+P)/2 | $1,375.27 | | | |
|  | IDegLira | 0.16902981 | 6366.55087 | 37665.2538 | > |
|  | Ideg |  |  |  |  |
| R | (M+Q)/2 | $1,375.25 | | | |
|  | IDegLira | 0.16902981 | 6365.49518 | 37659.0082 | > |
|  | Ideg |  |  |  |  |
| S | (M+R)/2 | $1,375.24 | | | |
|  | IDegLira | 0.16902981 | 6364.96734 | 37655.8854 | > |
|  | Ideg |  |  |  |  |
| T | (M+S)/2 | $1,375.23 | | | |
|  | IDegLira | 0.16902981 | 6364.70342 | 37654.3241 | < |
|  | Ideg |  |  |  |  |
| U | (S+T)/2 | $1,375.23 | | | |
|  | IDegLira | 0.16902981 | 6364.83538 | 37655.1048 | > |
|  | Ideg |  |  |  |  |
| V | (T+U)/2 | $1,375.24 | | | |
|  | IDegLira | 0.16902981 | 6364.90136 | 37655.4951 | > |
|  | Ideg |  |  |  |  |
| W | (T+V)/2 | $1,375.23 | | | |
|  | IDegLira | 0.16902981 | 6364.80239 | 37654.9096 | > |
|  | Ideg |  |  |  |  |
| X | (T+W)/2 | $1,375.23 | | | |
|  | IDegLira | 0.16902981 | 6364.7529 | 37654.6168 | > |
|  | Ideg |  |  |  |  |
| Y | (T+X)/2 | $1,375.23 | | | |
|  | IDegLira | 0.16902981 | 6364.72816 | 37654.4704 | < |
|  | Ideg |  |  |  |  |
| Z | (X+Y)/2 | $1,375.23 | | | |
|  | IDegLira | 0.16902981 | 6364.74053 | 37654.5436 | > |
|  | Ideg |  |  |  |  |
| AA | (Y+Z)/2 | $1,375.23 | | | |
|  | IDegLira | 0.16902981 | 6364.73435 | 37654.507 | ≈ |
|  | Ideg |  |  |  |  |

**Table S2** Outputs for binary search for further reduction in the annual cost of IDegLira, taking that of Lira as a reference.

| NO. | Group | ∆QALY | ∆cost | ICUR | Relationship with 3GDP |
| --- | --- | --- | --- | --- | --- |
| A | Baseline | $3,257.91 | | | |
|  | IDegLira | 0.05866645 | 8409.73299 | 143348.258 | > |
|  | Lira |  |  |  |  |
| B | A/2 | $1,628.95 | | | |
|  | IDegLira | 0.05866645 | -1731.0732 | -29507.04 | < |
|  | Lira |  |  |  |  |
| C | (A+B)/2 | $2,443.43 | | | |
|  | IDegLira | 0.05866645 | 3339.32991 | 56920.6091 | > |
|  | Lira |  |  |  |  |
| D | (B+C)/2 | $2,036.19 | | | |
|  | IDegLira | 0.05866645 | 804.128366 | 13706.7848 | < |
|  | Lira |  |  |  |  |
| E | (C+D)/2 | $2,239.81 | | | |
|  | IDegLira | 0.05866645 | 2071.72914 | 35313.697 | < |
|  | Lira |  |  |  |  |
| F | (C+E)/2 | $2,341.62 | | | |
|  | IDegLira | 0.05866645 | 2705.52952 | 46117.153 | > |
|  | Lira |  |  |  |  |
| G | (E+F)/2 | $2,290.71 | | | |
|  | IDegLira | 0.05866645 | 2388.62933 | 40715.425 | > |
|  | Lira |  |  |  |  |
| H | (E+G)/2 | $2,265.26 | | | |
|  | IDegLira | 0.05866645 | 2230.17923 | 38014.561 | > |
|  | Lira |  |  |  |  |
| I | (E+H)/2 | $2,252.54 | | | |
|  | IDegLira | 0.05866645 | 2150.95418 | 36664.129 | < |
|  | Lira |  |  |  |  |
| J | (H+I)/2 | $2,258.90 | | | |
|  | IDegLira | 0.05866645 | 2190.56671 | 37339.345 | < |
|  | Lira |  |  |  |  |
| K | (H+J)/2 | $2,262.08 | | | |
|  | IDegLira | 0.05866645 | 2210.37297 | 37676.953 | > |
|  | Lira |  |  |  |  |
| L | (J+K)/2 | $2,260.49 | | | |
|  | IDegLira | 0.05866645 | 2200.46984 | 37508.149 | < |
|  | Lira |  |  |  |  |
| M | (K+L)/2 | $2,261.29 | | | |
|  | IDegLira | 0.05866645 | 2205.4214 | 37592.551 | < |
|  | Lira |  |  |  |  |
| N | (K+M)/2 | $2,261.68 | | | |
|  | IDegLira | 0.05866645 | 2207.89719 | 37634.752 | < |
|  | Lira |  |  |  |  |
| O | (K+N)/2 | $2,261.88 | | | |
|  | IDegLira | 0.05866645 | 2209.13508 | 37655.8525 | > |
|  | Lira |  |  |  |  |
| P | (N+O)/2 | $2,261.78 | | | |
|  | IDegLira | 0.05866645 | 2208.51613 | 37645.3022 | < |
|  | Lira |  |  |  |  |
| Q | (O+P)/2 | $2,261.83 | | | |
|  | IDegLira | 0.05866645 | 2208.82561 | 37650.5773 | < |
|  | Lira |  |  |  |  |
| R | (O+Q)/2 | $2,261.86 | | | |
|  | IDegLira | 0.05866645 | 2208.98034 | 37653.2149 | < |
|  | Lira |  |  |  |  |
| S | (O+R)/2 | $2,261.87 | | | |
|  | IDegLira | 0.05866645 | 2208.74824 | 37649.2586 | < |
|  | Lira |  |  |  |  |
| T | (O+S)/2 | $2,261.88 | | | |
|  | IDegLira | 0.05866645 | 2208.94166 | 37652.5555 | < |
|  | Lira |  |  |  |  |
| U | (O+T)/2 | $2,261.88 | | | |
|  | IDegLira | 0.05866645 | 2209.03837 | 37654.204 | < |
|  | Lira |  |  |  |  |
| V | (O+U)/2 | $2,261.88 | | | |
|  | IDegLira | 0.05866645 | 2209.08672 | 37655.0282 | > |
|  | Lira |  |  |  |  |
| W | (U+V)/2 | $2,261.88 | | | |
|  | IDegLira | 0.05866645 | 2209.06255 | 37654.6161 | > |
|  | Lira |  |  |  |  |
| X | (U+W)/2 | $2,261.88 | | | |
|  | IDegLira | 0.05866645 | 2209.05046 | 37654.4101 | ≈ |
|  | Lira |  |  |  |  |
